# State of the art paper: Cardiac computed tomography of the left atrium in atrial fibrillation

**DOI:** 10.1016/j.jcct.2023.03.002

**Published:** 2023

**Authors:** Neil Bodagh, Michelle C. Williams, Keeran Vickneson, Ali Gharaviri, Steven Niederer, Steven E. Williams

**Affiliations:** a School of Biomedical Engineering and Imaging Sciences, King's College London, London, UK; b Centre for Cardiovascular Science, University of Edinburgh, Edinburgh, UK

**Keywords:** Atrial fibrillation, X-ray computed tomography, Critical pathways, Catheter ablation, Machine learning

## Abstract

The clinical spectrum of atrial fibrillation means that a patient-individualized approach is required to ensure optimal treatment. Cardiac computed tomography can accurately delineate atrial structure and function and could contribute to a personalized care pathway for atrial fibrillation patients. The imaging modality offers excellent spatial resolution and has been utilised in pre-, peri- and post-procedural care for patients with atrial fibrillation. Advances in temporal resolution, acquisition times and analysis techniques suggest potential expanding roles for cardiac computed tomography in the future management of patients with atrial fibrillation. The aim of the current review is to discuss the use of cardiac computed tomography in atrial fibrillation in pre-, peri- and post-procedural settings. Potential future applications of cardiac computed tomography including atrial wall thickness assessment and epicardial fat volume quantification are discussed together with emerging analysis techniques including computational modelling and machine learning with attention paid to how these developments may contribute to a personalized approach to atrial fibrillation management.

## 1 Introduction

Atrial fibrillation is a multifaceted, heterogenous disease requiring a patient-centered approach to manage symptoms, mitigate stroke risk, and optimize rhythm control where appropriate. Atrial fibrillation is conventionally classified according to a single domain - the temporal pattern of the arrhythmia. Whilst the use of classification systems has increased our understanding of atrial fibrillation to some extent, such an approach can overlook the importance of the interplay of factors related to the patient, their symptom severity, risk factors and the atrial electrophysiological substrate underpinning atrial fibrillation. Given these interactions, the management of atrial fibrillation necessitates the delivery of a coordinated patient-individualized care pathway to ensure optimal treatment. Whilst more research to explore the patient-specific pathophysiological basis of atrial fibrillation is necessary, the principles of patient-individualized care have been encapsulated in the 4S-AF scheme which aims to provide a structured, pathophysiology-based characterisation of a patient's atrial fibrillation comprising four domains - stroke risk, symptom severity, severity of atrial fibrillation burden and substrate severity.^1^ The importance of this approach has been underpinned by its implementation within European Society of Cardiology guidelines.^2^

Within this framework, computed tomography may provide useful information to guide atrial fibrillation management by enabling assessment of cardiac structure and function. Advances in computed tomography temporal and spatial resolution have strengthened its utility in the field of electrophysiology.^3^ In the context of atrial fibrillation catheter ablation, cardiac computed tomography has been applied in pre-, peri- and post-procedural care. In addition, the integration of cardiac computed tomography data into electroanatomic mapping systems can contribute to real-time guidance during ablation procedures. Software and hardware improvements mean that it is now possible to accurately assess the coronary arteries and atherosclerotic plaque in patients with atrial fibrillation and high heart rates.^4^ This information may have additive value in risk stratification and pre-procedure planning but is beyond the scope of this current article. Current research priorities into the use of computer tomography in guiding atrial fibrillation management include computational arrhythmia modelling based on computed tomography assessment of atrial structure, machine learning for atrial structure and electrical activity, assessment of left atrial wall thickness and scarring, and quantification of epicardial fat volume and attenuation.

**Fig. 1:**
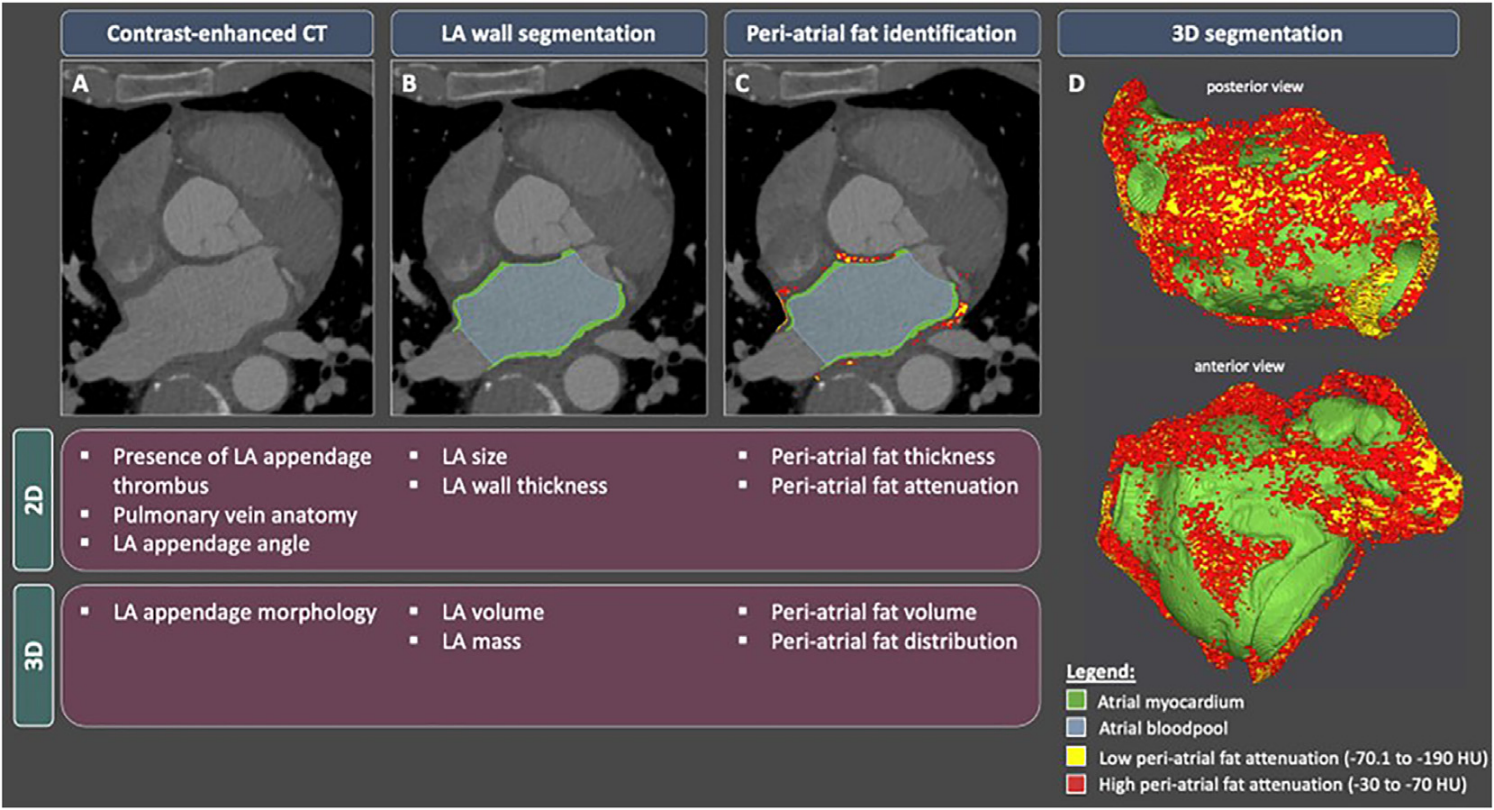
The current and potential value of computed tomography imaging in atrial fibrillation. (A) Contrast-enhanced computed tomography of the left atrium. (B) Semi-automatic segmentation of the left atrial wall and blood pool. (C) Identification of epicardial fat surrounding the left atrium where epicardial adipose tissue is defined as a hypodense layer with a density between −190 and −30 Hounsfield units. (D) Volumetric assessment of left atrium, peri-atrial fat and geographical distribution of peri-atrial fat and associated attenuation. CT, computed tomography; FAI, fat attenuation index; LA, left atrium; HU, Houndsfield units.

This review will discuss the current state-of-the-art for computed tomography in atrial fibrillation management together with potential future applications that form part of ongoing research (Fig. 1).

## 2 Current state-of-the art

### 2.1 Procedure planning

In atrial fibrillation, cardiac computed tomography has been used prior to direct current cardioversion, radiofrequency catheter ablation and left atrial appendage closure to investigate for contraindications, delineate anatomy and guide procedures.^3^ Recommendations on how to report pre-procedural cardiac computed tomography are provided in Table 1.

**Table 1:** Overview of the recommended items to report as part of a pre-procedural left atrial cardiac computed tomography scan for atrial fibrillation. Adapted from .^95^

	Recommendations for reporting a pre-procedural left atrial cardiac CT scan
General	Rhythm during acquisition
	Cardiac phase of imaging acquisition/reconstruction
	Contrast dose and radiation exposure
Left atrial appendage thrombus	Examine for a contrast filling defect, including on delayed phase images if performed
Left atrial appendage	Describe the left atrial appendage morphology
	Describe the left atrial appendage depth and length
	In assessments for left atrial appendage closure, report ostial and landing zone measurements
Atrial septum	Presence of atrial septal defect or patent foramen ovale
Left atrium	Provide absolute and indexed volumes
Pulmonary veins	Comment on the number, diameter, and morphology of pulmonary veins
	Comment on the presence/absence of stenoses
Pericardium	Examine for the presence of a pericardial effusion or pericardial thickening
Extra cardiac structures (relevant for atrial fibrillation ablation procedures)	Comment on the position of the esophagus, and in particular any parts that lie in close proximity to the left atrium
	Comment on the position of the phrenic nerves, if visualised, and in particular any parts that lie in close proximity to the left atrium

CT, computed tomography.

In the context of atrial fibrillation ablation procedures, pre-procedural atrial imaging can define patient-specific atrial anatomy.^5^ The integration of computed tomography data within electroanatomic mapping systems has previously been reported to shorten fluoroscopy and procedural times.^6^ However, contemporary electroanatomic mapping systems allow highly accurate reconstruction of pulmonary vein and left atrial anatomy without the need for pre-procedural cross-sectional imaging.^7^ In a recent study, DiCori et al., found no improvement in procedure duration or fluoroscopy time when pre-procedural computed tomography was used prior to ablation,^8^ which contrasts with previous analyses of the role of image integration in electroanatomic mapping procedures.^9^ DiCori et al. also found that the cumulative radiation dose was higher in the computed tomography group. These findings demonstrate the ongoing uncertainty that exists regarding the role of pre-procedural cross-sectional imaging in general and computed tomography imaging particularly when used purely for the assessment of atrial anatomy.

Prior to percutaneous left atrial appendage occlusion, pre-procedural imaging is utilised to guide left atrial appendage occluder device selection and sizing, and to facilitate procedure planning.^10^ Pre-procedural transesophageal echocardiography has frequently been used for this purpose. However, transesophageal echocardiography is a semi-invasive procedure which can cause oropharyngeal, oesophageal and/or gastric trauma, requires sedation or anaesthesia and presents additional clinical service demands.^11^ Computed tomography can provide a comprehensive three-dimensional assessment of left atrial appendage anatomy and may be of added benefit for patients undergoing left atrial appendage closure. Eng et al., have also shown that cardiac computed tomography may offer advantages in terms of device selection accuracy and procedural efficiency.^12^ They performed a randomized trial comparing pre-procedural computed tomography with transesophageal echocardiography for left atrial appendage occlusion planning and found that computed tomography was associated with improvements in device selection accuracy and shorter procedure times. Following left atrial appendage occlusion, computed tomography can also be utilized to evaluate for complications including incomplete left atrial appendage closure, peri-device leak, and device-related thrombus.^13^

### 2.2 Detection of left atrial appendage thrombus

In patients with sub-therapeutic anticoagulation (missed anticoagulant doses or short duration of therapy), investigation for left atrial appendage thrombi prior to direct current cardioversion or atrial instrumentation is required.^14^ Whilst transesophageal echocardiography has conventionally been used for this purpose, cardiac computed tomography can assess for the presence of left atrial appendage thrombus non-invasively and has similar diagnostic accuracy to transesophageal echocardiography.^15^ In a pooled meta-analysis of ten studies, Aimo et al. demonstrated that cardiac computed tomography has a sensitivity and specificity of 97% and 94% respectively for the detection of left atrial appendage thrombi, with an area under the curve of 0.99.^15^ Cardiac computed tomography therefore provides an alternate modality for investigation of left atrial appendage thrombi, which is of particular importance when transesophageal echocardiography is contraindicated or unavailable.

Computed tomography can be used to differentiate between left atrial appendage thrombus and slow blood flow by comparing early arterial and delayed phase images.^3^ On the early scan both appear as filling defects, whereas on the delayed phase thrombus is characterized by a persistent filling defect and slow blood flow is characterised by progressive contrast opacification. Hur et al., demonstrated that computed tomography could be used to identify left atrial appendage thrombus with good diagnostic accuracy compared to transesophageal echocardiography (sensitivity 100%, specificity 98%).^16^ Interestingly, spontaneous echo contrast in the left atrial appendage has been identified as an independent risk factor for thromboembolic events^17^ and further research is needed to understand the clinical implications of slow flow in the left atrial appendage. Radiomic analysis and dual energy imaging have both been proposed as new ways to identify left atrial appendage thrombus, which may reduce contrast dose or the requirement for additional imaging.^18^^,^^19^

### 2.3 Characterization of left atrial appendage morphology

**Fig. 2:**
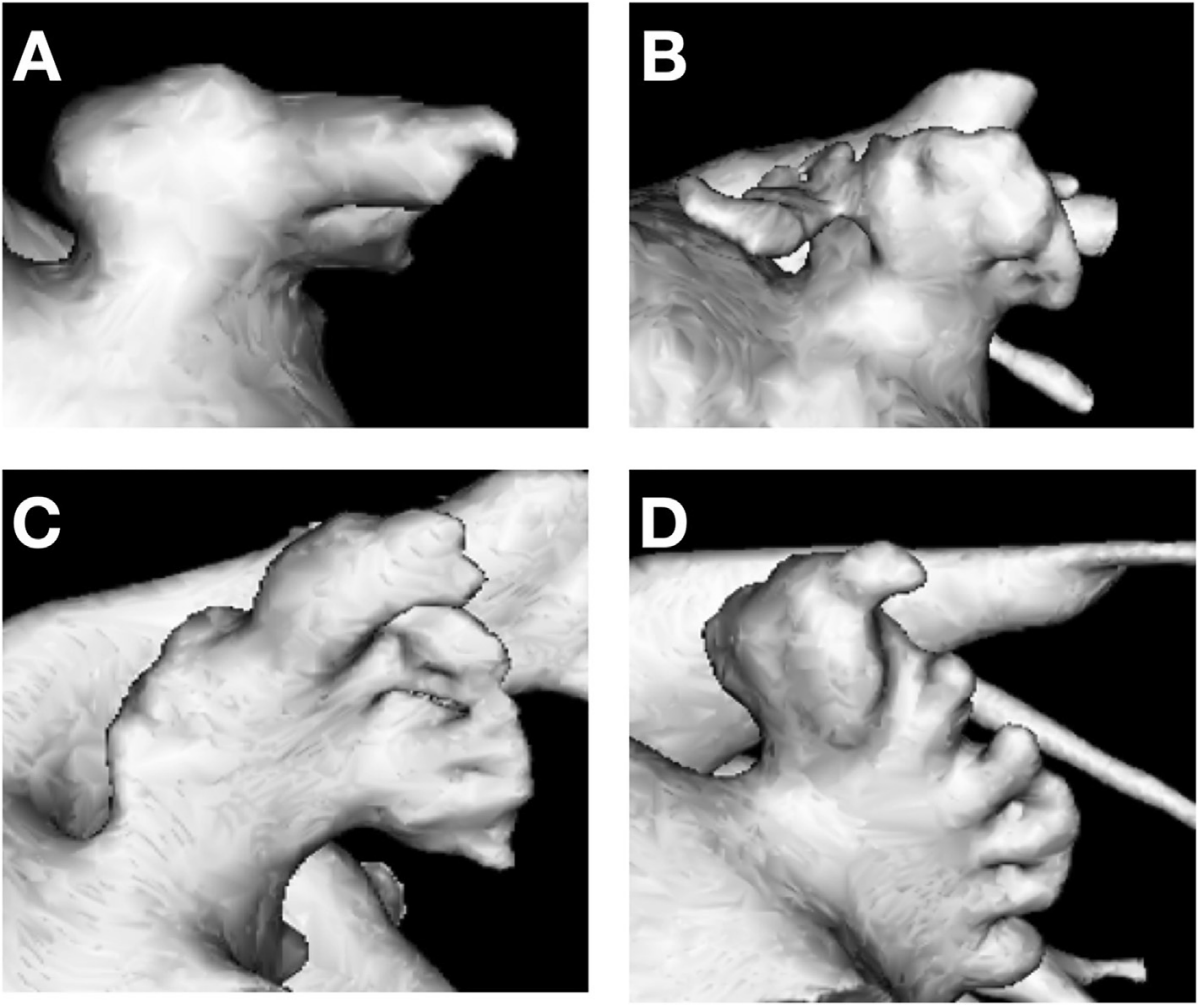
Left atrial appendage morphology in patients from the SCOT-HEART study. A – “windsock”, B – “chicken wing”, C – “cactus”, D – “cauliflower”.

**Fig. 3:**
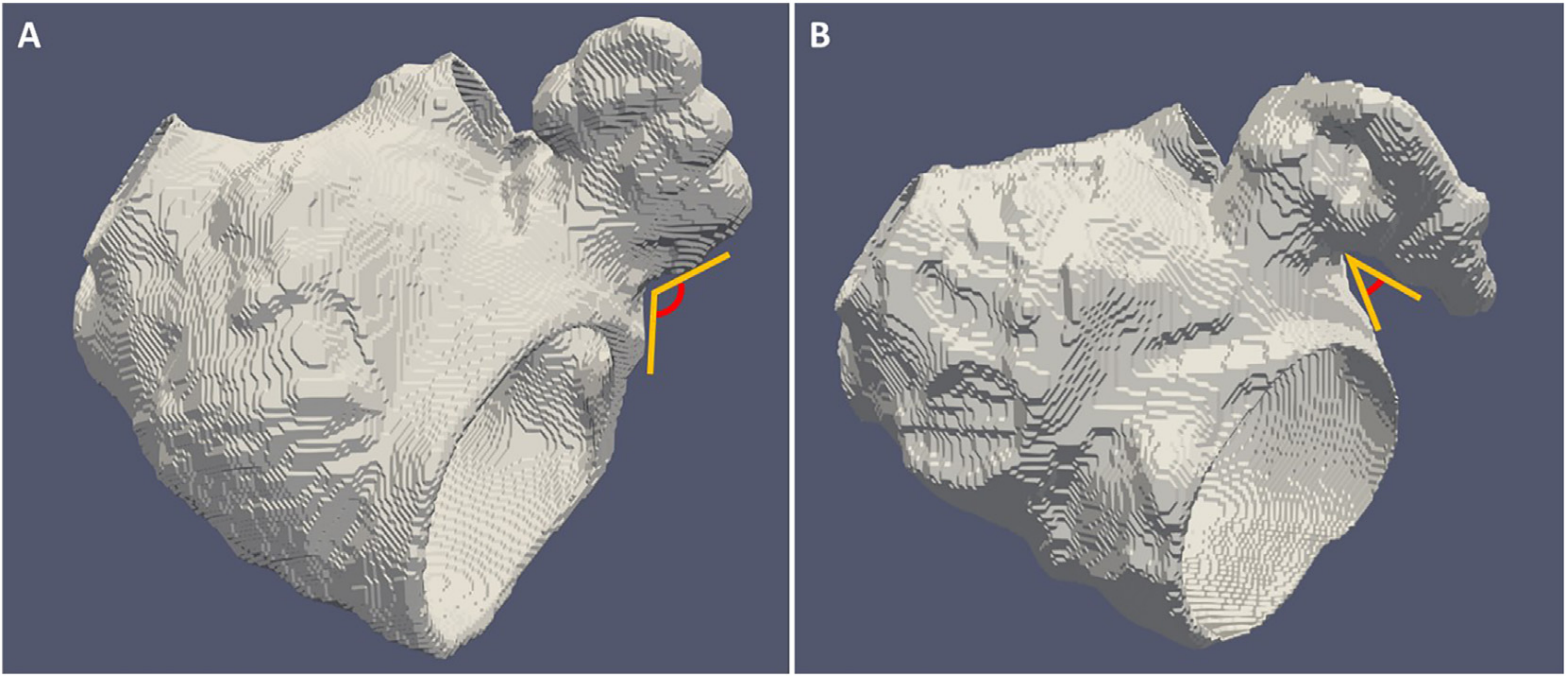
Image demonstrating a left atrial appendage classification system based on the angle of the bend. The image on the left side shows a high risk left atrial appendage (LAA-H) with ≥90° angle bend (A) and the image on the right side shows a low risk left atrial appendage (LAA-L) with <90° angle bend (B).

Cardiac computed tomography can be used to accurately characterize left atrial appendage morphology (Fig. 2). Left atrial appendage morphology has been correlated with stroke risk in patients with atrial fibrillation in several studies.^20^, ^21^, ^22^ DiBiase et al. characterized the left atrial appendage according to four morphologies (cactus, chicken wing, windsock, and cauliflower).^20^ The chicken wing morphology was associated with a lower risk of embolic events after controlling for comorbidities and the Congestive heart failure, Hypertension, Age, Diabetes and Stroke (CHADS_2_) score. In a recent simulation study by Qureshi et al., it has been demonstrated that left atrial appendage morphology may affect coagulation dynamics in blood flow during sinus rhythm and atrial fibrillation, and subsequent risk of thrombus formation.^23^ The chicken wing morphology was found to be most effective at slowing thrombus formation time in atrial fibrillation providing a potential mechanistic explanation for the observed lower embolic risk associated with a chicken wing morphology. However, the reliability of the classification system defined by DiBiase et al., remains unclear and there is no current consensus regarding the exact definitions of the morphologies which have instead been assigned based on visual assessment of appendage appearances. This has led to the development of a newer classification system based on the angle of the bend in the left atrial appendage (Fig. 3).^24^ Larger multi-center studies are required to validate the ability of this and other new classification systems to predict stroke risk. In the future, knowledge of a patient's left atrial appendage morphology could potentially be used in conjunction with other clinical information to guide decision making pertaining to anticoagulation therapy in patients with atrial fibrillation.

### 2.4 Pulmonary vein anatomy

Prior to radiofrequency catheter ablation of the pulmonary veins in atrial fibrillation, an awareness of a patient's left atrial and pulmonary vein anatomy can enable accurate targeting and planning through the measurement of ostial diameter, characterization of variants in pulmonary vein anatomy such as accessory pulmonary veins or common pulmonary vein ostia, and the identification of anomalies such as anomalous pulmonary venous return, pulmonary vein occlusion or the presence of a persistent left superior vena cava.^25^ Cardiac computed tomography offers excellent spatial resolution and is considered the gold standard in assessing the diameter, morphology, and anatomy of the pulmonary veins.^26^

Variations in pulmonary vein anatomy are common and can be easily assessed with computed tomography.^27^ Some studies have shown that isolation of supernumerary veins may contribute to procedural success in atrial fibrillation ablation.^28^ However, other studies have demonstrated that outcomes following cryoballoon therapy or radiofrequency ablation are unaffected by variations in pulmonary vein anatomy.^29^ Computed tomography imaging may be able to predict the likely difficulty of pulmonary vein isolation during cryoballoon ablation for atrial fibrillation. Ang et al. found that a larger maximum to minimum ostial diameter detected by computed tomography (termed the eccentricity index) was associated with a greater difficulty in isolating the left inferior pulmonary vein during cryoballoon ablation.^30^ Translation of these observations into prediction of long-term procedural outcomes or to guide selection of ablation modalities requires further investigation.

### 2.5 Pulmonary vein stenosis

Pulmonary vein stenosis is a recognized complication of atrial fibrillation ablation close to or within the pulmonary vein ostia. Symptoms typically take weeks to months to manifest and include shortness of breath, dry cough and, in some instances, chest pain.^31^ Pulmonary vein occlusion can lead to venous infarction and pulmonary hypertension.^32^ In cases of pulmonary vein stenosis, cardiac computed tomography can demonstrate direct imaging findings such as an abrupt cut off or narrowing of a pulmonary vein as well as indirect imaging findings such as regional lung oedema, lung infarction or an increased blood/contrast transit time in the lung drained by the stenosed vein.^33^

### 2.6 Extra-cardiac structures

Injury to extra-cardiac structures is a recognized, but fortunately rare, complication of atrial fibrillation ablation. The esophagus lies adjacent to the posterior wall of the left atrium leaving it vulnerable to injury during radiofrequency catheter ablation.^34^ This can lead to the formation of atrio-esophageal fistula, a rare but potentially life-threatening complication of radiofrequency catheter ablation.^32^ Cardiac computed tomography can identify areas of the left atrium lying in direct proximity to the esophagus.^35^ Theoretically, this could allow titration of ablation energy delivery in atrial regions close to the esophagus to minimize the risk of esophageal injury. However, this has not been implemented in routine clinical practice as the use of pre-procedural computed tomography imaging is limited by the potential for intra-procedural esophagus movement.^36^ This has resulted in the development of real-time methods using modalities such as intracardiac echocardiography^37^ and mapping/ablation catheters^38^ to assess the oesophageal position during catheter ablation.

Methods to reduce risk of atrio-esophageal fistula formation such as active esophageal cooling^39^ and mechanical esophageal displacement^40^ in conjunction with pre- and intra-procedural esophageal imaging have also been of variable success. Signs and symptoms of this life-threatening complication are non-specific, and the time of onset is highly variable. Computed tomography imaging with contrast can rapidly and reliably diagnose atrio-esophageal fistula, with the visualisation of intravenous contrast passing from the left atrium into the esophagus, with other potential features including gas in the cardiac chambers, pericardial effusion, pneumomediastinum, left atrial wall thickening and adjacent fat stranding.^41^

The proximity of the right and left phrenic nerves with the right superior pulmonary vein and left atrial appendage roof respectively also leaves these structures vulnerable to injury during ablation.^42^ Imaging of the right cardiophrenic artery with cardiac computed tomography can locate the right phrenic nerve. Horton et al. showed that an approximated right phrenic nerve location within 10 ​mm of the right superior pulmonary vein conferred a higher risk of phrenic nerve injury.^43^ However, this technique is not routinely implemented in clinical practice as other techniques are used to minimize the risk of phrenic nerve injury such as immediate balloon deflation during cryoablation, pacing to identify phrenic nerve capture and intra-procedural diaphragmatic monitoring.^44^

## 3 The future

Advances in computed tomography image acquisition and analysis provide the opportunity for its role in atrial fibrillation assessment to expand. Techniques including computational modelling, artificial intelligence-based imaging, atrial wall thickness assessment and epicardial adipose tissue characterisation may enable an individualized precision-cardiology approach to atrial fibrillation management. This section summarizes these approaches and suggests areas where future research developments using computed tomography may address knowledge gaps in the management of atrial fibrillation.

### 3.1 Computational modelling of atrial structure and function

**Fig. 4:**
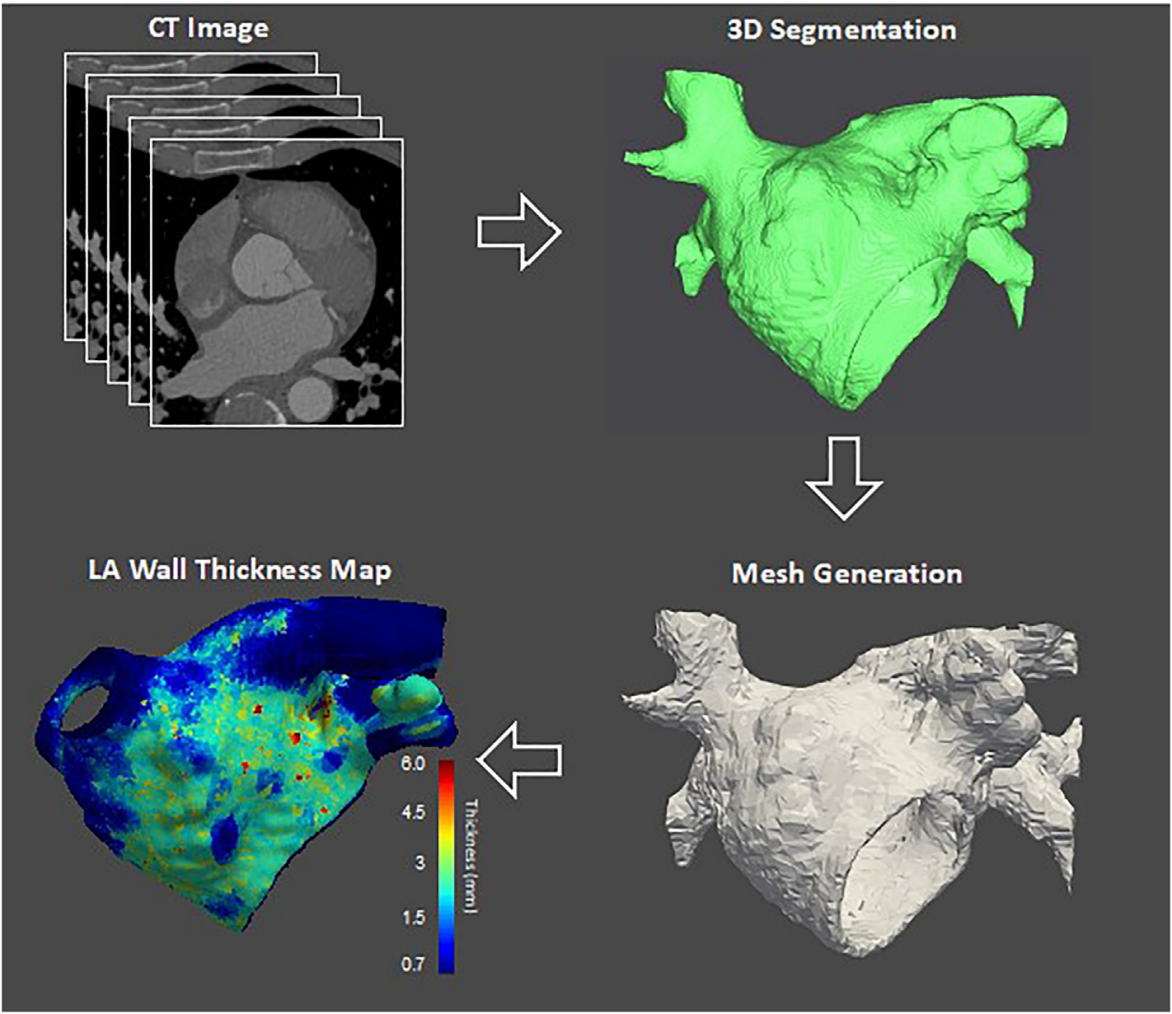
Atrial fibrillation simulation procedure - Integrating personalized left atrial anatomy to generate a left atrial wall thickness map for *in silico* atrial fibrillation simulations. CT, computed tomography; LA, left atrium.

The ability of cardiac computed tomography to accurately delineate atrial structure can enable the acquisition of complex three-dimensional human atrial geometry which can form the basis of computational organ-level atrial models (Fig. 4).^45^ These use information from cell-level scales and mathematical modelling of human action potential conduction to simulate electrical propagation in atrial fibrillation.^46^ The use of one-dimensional and two-dimensional tissue models can be used to investigate atrial fibre architecture and provide an understanding of how electrical conduction occurs within tissue.^47^

Computational models could be used to characterize an individual's substrate severity and lead to personalized atrial fibrillation ablation therapy. Hwang et al. used cardiac computed tomography to create atrial models which could be used to compare different ablation strategies for atrial fibrillation.^45^ Computed tomography was used to generate patient-specific three-dimensional left atrial geometry for finite element models for twenty patients and these were used to test five different virtual ablation strategies and compare outcomes. A small, randomized trial has subsequently demonstrated superiority of a computational modelling-based catheter ablation approach versus empirical catheter ablation with respect to the trial's primary endpoint of freedom from atrial tachyarrhythmia lasting longer than 30 ​s.^48^ It is promising that this approach has been shown to lead to a reduction in atrial fibrillation burden. However, further research is required to assess whether this translates into an improvement in clinical outcomes.

Computational modelling for personalized atrial fibrillation procedure planning is an area of active research, offering the potential to better understand arrhythmia pathophysiology and improve clinical arrythmia prevention, risk stratification and treatment.^49^ In the future we may be able to create patient-specific computational models which capture the mechanisms underpinning the initiation and maintenance of atrial fibrillation in individual patients and subsequently design pathophysiology-based treatment strategies. The Realistic Computational Electrophysiology Simulations for the Targeted Treatment of Atrial Fibrillation (ReCETT-AF) (ClinicalTrials.gov, #NCT05057507) study will aim to use computational modelling to define patient-specific mechanisms of atrial fibrillation.

**Fig. 5:**
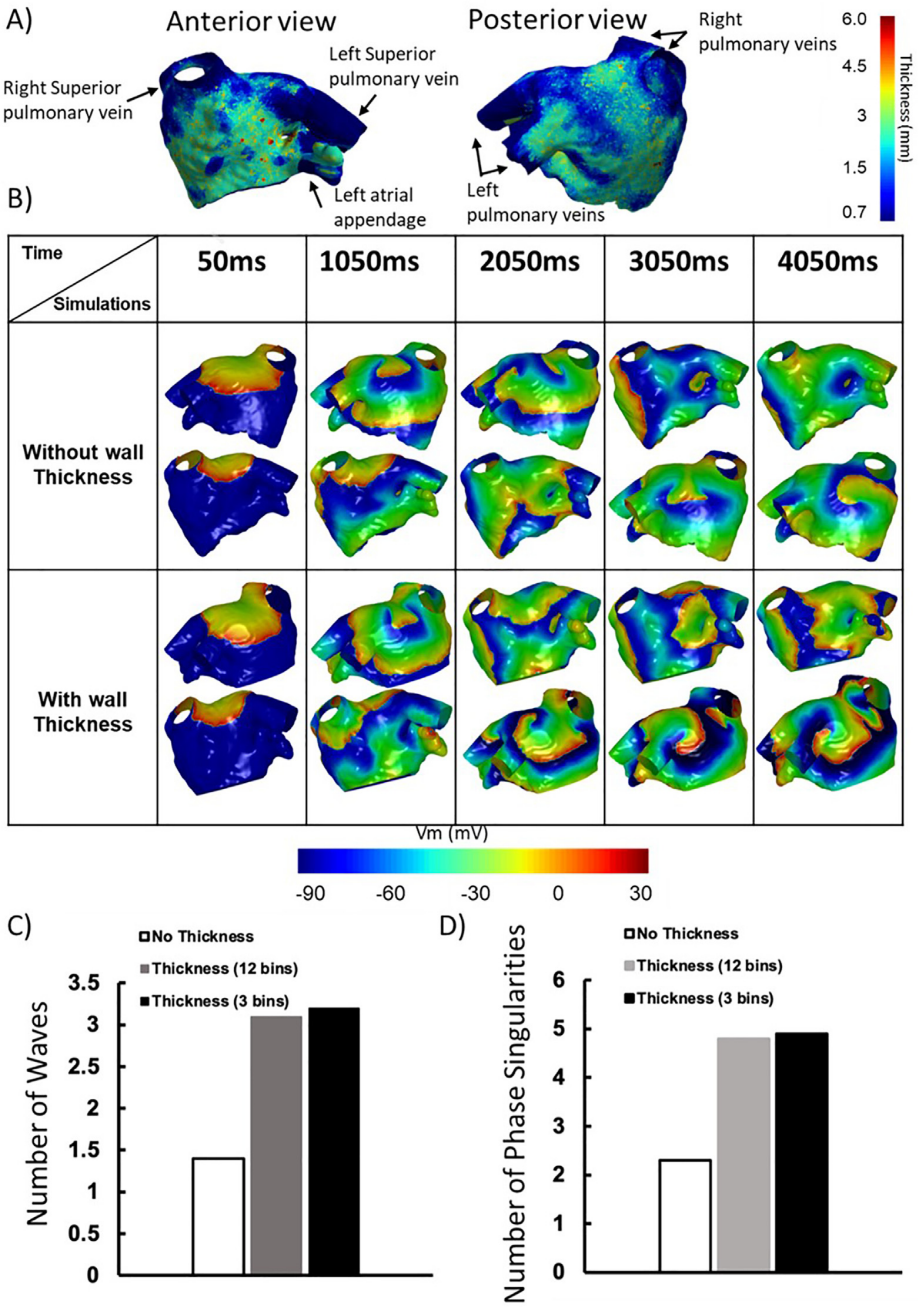
Computed tomography-based computational modelling evaluating the impact of changes in left atrial wall thickness on atrial fibrillation propagation. (A) Patient-specific computational model incorporating computed tomography-derived measurements of left atrial wall thickness. (B) Electrophysiology simulations showing patterns of wavefront propagation in computational models ""without wall thickness” and “with wall thickness” data. (C) and (D) Bar charts demonstrating the impact of left atrial wall thickness on the numbers of waves and phase singularities observed in atrial fibrillation simulations, with the atrial wall represented with 12 bins (0.5 ​mm width) or 3 bins (2 ​mm width).

Future research developments using computational models will require three-dimensional anatomical geometry and cardiac computed tomography is therefore likely to play a role in the generation of these models. A key advantage of cardiac computed tomography in this setting is the high spatial resolution of image acquisition which enables accurate anatomical assessment of wall thickness and left atrial appendage morphology. Previous studies using magnetic resonance imaging to evaluate the role of computational modelling in atrial fibrillation therapy have been unable to include variations in left atrial wall thickness or appendage morphology.^50^ Interestingly, variations in left atrial wall thickness have been correlated with wave dynamics in atrial fibrillation,^51^ whilst the left atrial appendage is a frequent location for re-entrant drivers sustaining atrial fibrillation.^52^ Computational models which include wall thickness and atrial appendage morphology may identify variations in atrial fibrillation complexity which are not evident when these parameters are excluded from simulations (Fig. 5), underlining the importance of considering detailed patient-specific atrial anatomy when investigating the mechanisms sustaining atrial fibrillation.

### 3.2 Machine learning

Machine learning is likely to be beneficial for personalized atrial fibrillation care in the future.^53^ In atrial fibrillation screening pathways, machine learning has the potential to improve stroke prediction tools and optimise treatment strategies.^54^ The potential value of machine learning-based computed tomography in atrial fibrillation screening has been demonstrated by Aquino et al.,^55^ The authors found that a deep learning algorithm enabled automated measurement of left atrial volume on non-contrast lung cancer screening scans and that this measurement may be associated with an increased risk of new-onset atrial fibrillation within five years. Whilst further studies are required to delineate the precise link between automated computed tomography measurements and risk of atrial fibrillation, such a study demonstrates the potential for machine learning to enable population screening on scans performed for other purposes. This may enable the implementation of technologies to facilitate screening, diagnosis, and risk stratification in atrial fibrillation in the future.^14^

Improved image quality with machine learning-based reconstruction algorithms may improve the ability to assess thin structures such as the atrial wall.^56^ Furthermore, the use of machine learning-based algorithms can enable fast, fully automated quantification of left atrial volume and function with similar levels of accuracy in comparison to manual quantification by an expert radiologist.^57^ The use of such algorithms has the potential to increase efficiency, reduce interobserver variability and enable accurate, reproducible atrial assessment.

The ability of cardiac computed tomography to characterise atrial structure may prove to be of prognostic importance as structural parameters such as left atrial volume^58^ and atrial sphericity^59^ have been shown to predict recurrence of arrhythmia after radiofrequency catheter ablation. However, the relationship between these biomarkers and atrial fibrillation recurrence is complex. Machine learning may help to decipher the combined impact of these biomarkers. Atta-Fosu et al. developed a machine learning model based on statistical shape differentiation to identify regional differences in left atrial shape on pre-ablation computed tomography between patients who did and did not have atrial fibrillation recurrence post ablation.^60^ In a retrospective study of 68 patients, the authors identified surfaces of interest on atlases of atrial models. These were then used to show areas of the atrial surface that differed in patients with and without atrial fibrillation recurrence. The authors found that differences in shape around the pulmonary vein ostia and left atrial appendage were predictive of recurrence and subsequently highlighted the requirement to perform future studies to precisely identify the exact types of shape changes associated with atrial fibrillation recurrence. Machine learning has also been applied in computed tomography to non-invasively predict left atrial wall stress with higher left atrial wall stress associated with increased atrial fibrillation recurrence post-catheter ablation.^61^ In the future, pre-procedural radiomic features may be used to predict the likelihood of post-ablation recurrence in conjunction with clinical information to guide decision making regarding treatment.

### 3.3 Atrial wall thickness assessment

Changes in the structure and electrical behavior of the left atrium are associated with conditions which predispose to atrial fibrillation, although it is also recognized that these changes may occur in response to atrial fibrillation itself.^62^ Cardiac computed tomography can be used to measure left atrial wall thickness (Fig. 1B) and has been used to demonstrate that left atrial wall thickness is increased in atrial fibrillation.^63^ The high spatial resolution of computed tomography has led to the modality emerging as the optimal imaging tool for left atrial wall thickness assessment.^62^ We have proposed a method for left atrial wall thickness measurement using the Laplace equation and demonstrated that this method can accurately measure left atrial wall thickness across the entire left atrium.^64^ The method initially comprises computed tomography imaging from which the left atrium is segmented, and the Laplace equation is then subsequently solved over a computational model derived from the segmented left atrium to enable left atrial wall thickness to be calculated.

Furthermore, we have developed reference values for left atrial wall thickness measurements on computed tomography^65^ using coronary computed tomography angiography imaging from 193 asymptomatic patients recruited to the Burden of atherosclerotic plaques in twins – Genetic Loci and the Burden of Atherosclerotic Lesions (BUDAPEST GLOBAL) study.^66^ We found that the overall left atrial median wall thickness was 1.45 ​mm and that reference ranges varied depending on location. The ranges obtained are presented in Table 2.

**Table 2:** Reference ranges for computed tomography-derived left atrial wall thickness tabulated and summarized from.^65^

Left atrial location	Left atrial wall thickness (mm)		
	Median	Range	5–95% range
Inferior portion of posterior wall	1.14	0.57–5.99	0.72–2.71
Roof	1.55	0.56–15.19	0.70–3.72
Anterior aspect	1.65	0.71–5.76	0.83–2.99
Superior portion of posterior wall	1.73	0.58–11.79	0.84–2.89
Septum	2.00	0.83–6.49	1.07–3.44

Studies have also been performed to examine for an association between left atrial wall thickness measurements and atrial fibrillation ablation success rates. Zuo et al.*,* measured left atrial wall thickness at two locations (roof and floor) using computed tomography in 100 persistent atrial fibrillation patients.^67^ Catheter ablation was subsequently performed, and the degree of atrial remodelling was inferred according to response to pulmonary vein isolation or complex fractionated atrial electrogram ablation, and categorized as weak, mild, or strong ‘maintenance substrate’. The authors found that areas of stronger atrial maintenance substrate correlated with areas of increased left atrial wall thickness. Whilst this finding is of interest, the lack of electrophysiological characterization of atrial remodelling, which is instead inferred from procedural response, precludes firm conclusions regarding the relationship between atrial wall thickness and atrial remodelling. Furthermore, Oh et al.*,* measured thickness around the pulmonary vein antra in patients undergoing repeat ablation procedures and found that antral wall thickness was higher in areas of pulmonary vein reconnection.^68^ In contrast to these findings, Boussoussou et al.*,* found no association between left atrial wall thickness measurements and pulmonary vein isolation success rates.^69^ Further studies are warranted to elucidate the relationship between left atrial wall thickness and catheter ablation success rates.

The use of computed tomography to assess atrial wall thickness and provide non-invasive substrate determination may enable us to identify areas where the risk of recurrence is potentially higher and personalize therapy to improve outcomes. The utility of computed tomography-derived left atrial wall thickness in guiding catheter ablation approach has been demonstrated by Teres et al., who found that personalized ablation approaches adapted to left atrial wall thickness allowed pulmonary vein isolation with lower radiofrequency delivery, fluoroscopy, and procedure times.^70^ Whilst further study is required, left atrial wall thickness measurements show promise for guiding treatment decision-making regarding patient selection and approach for catheter ablation.

### 3.4 Epicardial fat and atrial fibrillation

Cardiac computed tomography enables a three-dimensional assessment of the volume, thickness, and attenuation of the epicardial adipose tissue (Fig. 1C).^71^ Some studies have assessed the entire epicardial adipose tissue whereas others have focussed on the epicardial adipose tissue surrounding the left atrium, between the myocardium and pericardium. The observation that increased epicardial adipose tissue volume may be associated with an increased risk of cardiovascular events has prompted considerable interest in the study of epicardial adipose tissue.^72^ Epicardial adipose tissue is typically visualised as a hypodense layer with a density between −190 and −30 Hounsfield units,^73^ although other studies have defined epicardial adipose tissue with a range between −195 and −45 Hounsfield units.^74^ Methods to assess epicardial adipose tissue using computed tomography currently vary and future studies are warranted to define standardised reference levels.

Several studies have shown that patients with atrial fibrillation have increased epicardial adipose tissue thickness, mass and volume compared with patients without atrial fibrillation (Table 3). A meta-analysis by Wong et al., demonstrated that higher epicardial adipose tissue volumes are associated with the presence of atrial fibrillation to a greater extent than other adipose tissue deposits such as abdominal and overall adiposity.^75^ The association between epicardial adipose tissue and atrial fibrillation appears to be independent of markers of adipose tissue type such as body mass index and body surface area.^76^ Interestingly, it has been demonstrated that epicardial adipose tissue volume appears to be independent of left atrial size in patients with atrial fibrillation.^77^ Increased epicardial adipose tissue volume has been demonstrated to be an independent predictor of the subsequent development of atrial fibrillation^76^^,^^78^ and stroke.^79^ In patients with COVID-19, epicardial adipose tissue volume was associated with a higher risk of developing new-onset atrial fibrillation.^80^ Several studies have assessed the association between epicardial adipose tissue volume and recurrence of atrial fibrillation after ablation, but results have been inconsistent (Table 3). Statin therapy may reduce the volume of epicardial adipose tissue, thus highlighting a potential preventative treatment.^81^

**Table 3:** Research studies assessing the impact of computed tomography assessment of epicardial adipose tissue on atrial fibrillation.

Author	Year	N	AF	EAT	Findings
**Amount of EAT in AF patients versus controls**					
Van Rosendael^96^	2022	300	67%	Mass	Posterior LA mass was higher in AF
Gaibazzi^85^	2021	160	50%	Mass	Not higher in AF, when indexed for LA size
Zhu^97^	2021	2042	8.5%	Volume	Higher in AF, independent of BMI
Oba^98^	2018	316	65%	Volume	Higher in AF, indexed to BSA
Yorgun^99^	2015	494	61%	Thickness	Higher in AF
Tsao^100^	2011	98	65%	Volume	Higher in AF
**Amount of EAT and AF recurrence post ablation**					
Jian^101^	2022	337	100%	Thickness	Independent predictor of recurrence
Yang^87^	2022	608	100%	Volume	Not associated with recurrence
Beyer^88^	2021	732	100%	Volume	Higher in those with recurrence
Hammache^89^	2021	389	100%	Volume	LA-EAT was not associated with recurrence
Vroomen^102^	2019	85	100%	Volume	Periatrial EAT was associated with recurrence, indexed to BSA
Sanghai^103^	2018	274	100%	Area	LA-EAT was an independent predictor of recurrence
Maeda^104^	2018	218	100%	Volume	Independent predictor of recurrence
Masuda^105^	2015	53	100%	Volume	LA but not total EAT was a predictor of recurrence
Stojanovska^106^	2015	231	73%	Volume	Independent predictor of recurrence
Kocyigit^107^	2015	249	100%	Thickness	Periatrial but not total EAT was a predictor of recurrence
Tsao^100^	2011	98	65%	Volume	Higher in those with recurrence.
**Amount of EAT and development of AF**					
Bos^76^	2017	1990	0%	Volume	Independent predictor.
Nakanishi^78^	2012	279	0%	Volume	LA-EAT was an independent predictor.
Mahabadi^108^	2014	3467	0%	Volume	Not predictive of AF development, independent of LA size
**EAT Attenuation and AF presence**					
Gaibazzi^85^	2021	160	50%	Attenuation	Mean LA-EAT was higher in AF (−69.15HU) versus non-AF (−76.82HU); (p ​< ​0.0001)
Kusayama^86^	2016	64	50%	Attenuation	Mean LA-EAT density higher in AF (108.61 ​± ​6.7HU) versus non-AF (111.6 ​± ​5.5HU); (p ​= ​0.02). This finding was independent of risk factors
**EAT attenuation and recurrence post ablation**					
Yang^87^	2022	608	100%	Attenuation	LA-EAT associated with recurrence (mean LA-EAT density −76.16 ​± ​4.11HU in the recurrence group versus −78.83 ​± ​3.81HU in the non-recurrence; p ​< ​0.001).
Mahdiui^74^	2021	460	100%	Attenuation	Independent predictor of recurrence. Patients were split into groups with higher attenuation (>-96.4HU) or lower attenuation (≤96.4HU). Patients in the higher attenuation group were more likely to have AF recurrence.
Beyer^88^	2021	732	100%	Attenuation	Associated with recurrence (mean LA-EAT density −69.1 HU in the recurrence group versus −67.5 HU in the non-recurrence group; p ​= ​0.001)
Hammache^89^	2021	389	100%	Attenuation	Not associated with recurrence (mean LA-EAT density −93.7 ​± ​4.3HU in the recurrence group versus −93.4 ​± ​6.0HU in the non-recurrence group, p ​= ​0.556)

AF, atrial fibrillation; BSA, body surface area; CABG, coronary artery bypass graft; EAT, epicardial adipose tissue; HU, Hounsfield units; N, number; LA, left atrium .

Recently, assessment of the pericoronary adipose tissue attenuation has gained attention due to its association with subsequent cardiac events.^82^ Increases in pericoronary adipose tissue attenuation have also been associated with atrial fibrillation recurrence post-ablation^83^ and this may reflect inflammation, which has been implicated in the pathogenesis of atrial fibrillation.^84^ The attenuation of epicardial adipose tissue, particularly that around the left atrium, can be also assessed and has been found to be higher in patients with atrial fibrillation,^85^^,^^86^ independent of left atrial size, body mass index, and clinical factors (Table 3). Several studies have also shown that patients with higher epicardial adipose tissue attenuation have increased atrial fibrillation recurrence post ablation, independent of left atrial volume, body mass index, echocardiographic parameters, and clinical factors.^74^^,^^87^^,^^88^ However, Hammache et al., showed that neither total epicardial adipose tissue volume nor left atrial epicardial adipose tissue attenuation were associated with recurrence of atrial fibrillation post ablation in 389 paroxysmal atrial fibrillation patients who were followed up for one year.^89^

Notably these studies have considered only single-plane analysis or peri-atrial fat attenuation limited to the posterior left atrium. Furthermore, cut-off values to accurately define abnormal epicardial adipose tissue attenuation have not been defined and vary between research studies. Further mechanistic studies are therefore needed to study the role of global peri-atrial fat and fat attenuation in atrial fibrillation pathogenesis.

In a study by Zhang et al., it has been shown that the radiomic characteristics of epicardial adipose tissue may improve the identification of patients who have atrial fibrillation compared to quantification of epicardial adipose tissue volume.^90^ The authors demonstrated that a radiomics analysis based on machine learning identified atrial fibrillation with an area under the curve of 0.92 (95% confidence interval 0.84–1.00), higher than the area under the curve of 0.73 (95% confidence interval 0.61–0.86) for a model based on epicardial adipose tissue volume. Epicardial adipose tissue radiomic signatures may also predict atrial fibrillation recurrence following catheter ablation.^91^ Further studies are therefore warranted to ascertain the precise role of epicardial adipose tissue radiomic analysis in the diagnosis and management of atrial fibrillation.

At present it is unknown whether changes in epicardial adipose tissue are a cause or consequence of atrial fibrillation. Epicardial adipose tissue, which contains ganglionated plexi, actively secretes cytokines and adipokines which may lead to inflammation and atrial remodelling. The secretion of pro-fibrotic factors by epicardial adipose tissue may contribute to atrial myocardial fibrosis and this may act as a substrate for atrial fibrillation.^92^ Epicardial adipose tissue is also a source of inflammatory mediators^93^ and it is well recognized that inflammation and atrial fibrillation are linked.^84^ Furthermore, epicardial adipose tissue is densely populated with sympathetic and parasympathetic nerve fibers.^94^ Thus, it is possible that epicardial adipose tissue may modulate autonomic effects thereby contributing to the pathogenesis of atrial fibrillation. In the future, quantification of epicardial fat with cardiac computed tomography may improve the management of patients with or at risk of atrial fibrillation. Several studies are currently being performed to evaluate the prognostic role of epicardial adipose tissue in atrial fibrillation and to examine the impact of atrial fibrillation treatment modalities on computed tomography-derived epicardial adipose tissue measurements (Table 4).

**Table 4:** In progress atrial fibrillation studies using computed tomography-derived epicardial adipose tissue measurements.

Study name	ClinicalTrials.gov Identifier	Description
Contribution of Computed Tomography and Cardiac-MRI in Atrial Fibrillation Ablation (CTStrain-AF)	NCT04281329	Prospective cohort study aiming to examine the impact of CT-measured epicardial adipose tissue fat volume and MRI-measured left atrial strain on rates of AF recurrence at 1 year after ablation (following a 3-month blanking period).
VOLTage Mapping in Atrial Fibrillation (VOLT-AF)	NCT04832646	Retrospective cohort study which aims to define a new method for quantifying left atrial voltage using a total energy map instead of a peak-to-peak amplitude map. The study will also examine whether there is a correlation between pre-ablation CT-measured epicardial adipose tissue deposits and atrial conduction velocity slowing.
Prospective Evaluation of CT Guided Ablation of Cardiac Ganglionated Plexi	NCT04642976	Prospective cohort study aiming to examine the feasibility of CT and high frequency simulation-guided ganglionated plexi mapping and ablation. The investigators aim to develop a method to enable CT to guide ganglionated plexi localization in patients undergoing AF ablation procedures. The primary outcome of the study will be the correlation between high frequency signals and CT-identified epicardial adipose tissue.
Liraglutide Effect in Atrial Fibrillation (LEAF)	NCT03856632	Randomized trial which aims to compare liraglutide plus risk factor modification versus risk factor modification alone on the change in size of left atrial epicardial adipose tissue at 3 months (assessed using CT).
LOSE-AF: Can Weight Loss Help Patients With Atrial Fibrillation? (LOSE-AF)	NCT03713775	Open-label randomized controlled trial which will aim to examine whether a weight loss programme with meal replacement and behavioural support can reduce AF recurrences and improve physical performance compared to usual care. As a secondary outcome, the fat attenuation index of epicardial adipose tissue will be measured 8 months after randomisation to either study arm.

CT, computed tomography; MRI, magnetic resonance imaging; AF, atrial fibrillation.

## 4 Conclusion

Cardiac computed tomography can provide important anatomical information for patients with atrial fibrillation and has been used in the pre-, peri- and post-procedural stages of management. New techniques such as computational modelling and machine learning are improving our understanding of the development of atrial fibrillation and responses to therapy. In the future information such as atrial structure, atrial wall thickness, and epicardial adipose tissue from computed tomography may be used to provide a personalized approach for the management or prevention of atrial fibrillation.

## Disclosures

MCW has given talks sponsored by Canon Medical Systems, Siemens Healthineers and Novartis. SN receives research funding from Siemens for computed tomography image analysis. SEW has consulting agreements with EPD/Phillips, GSK and Biosense Webster.
